# The Role of Siglec-G on Immune Cells in Sepsis

**DOI:** 10.3389/fimmu.2021.621627

**Published:** 2021-02-23

**Authors:** William Royster, Ping Wang, Monowar Aziz

**Affiliations:** ^1^Center for Immunology and Inflammation, The Feinstein Institutes for Medical Research, Manhasset, NY, United States; ^2^Elmezzi Graduate School of Molecular Medicine, Manhasset, NY, United States; ^3^Department of Surgery, Donald and Barbara Zucker School of Medicine at Hofstra/Northwell, Manhasset, NY, United States

**Keywords:** sialic acid-binding immunoglobulin-type lectin-G (Siglec-G), B-1a cells, B cell receptor (BCR), TLR, damage-associated molecular patterns (DAMPs), extracellular cold-inducible RNA-binding protein (eCIRP), inflammation, sepsis

## Abstract

Sepsis is a life-threatening clinical syndrome that results from an overwhelming immune response to infection. During sepsis, immune cells are activated by sensing pathogen-associated molecular patterns and damage-associated molecular patterns (DAMPs) through pattern recognizing receptors (PRRs). Regulation of the immune response is essential to preventing or managing sepsis. Sialic acid-binding immunoglobulin-type lectin-G (Siglec-G), a CD33 group of Siglec expressed in B-1a cells and other hematopoietic cells, plays an important immunoregulatory role. B-1a cells, a subtype of B lymphocytes, spontaneously produce natural IgM which confers protection against infection. B-1a cells also produce IL-10, GM-CSF, and IL-35 to control inflammation. Sialic acids are present on cell membranes, receptors, and glycoproteins. Siglec-G binds to the sialic acid residues on the B cell receptor (BCR) and controls BCR-mediated signal transduction, thereby maintaining homeostasis of Ca^++^ influx and NFATc1 expression. Siglec-G inhibits NF-κB activation in B-1a cells and regulates B-1a cell proliferation. In myeloid cells, Siglec-G inhibits DAMP-mediated inflammation by forming a ternary complex with DAMP and CD24. Thus, preserving Siglec-G’s function could be a novel therapeutic approach in sepsis. Here, we review the immunoregulatory functions of Siglec-G in B-1a cells and myeloid cells in sepsis. A clear understanding of Siglec-G is important to developing novel therapeutics in treating sepsis.

## Introduction

Inflammation refers to the body’s immune response to foreign particles. Sepsis is characterized as life threatening organ dysfunction caused by a dysregulated host response to infection (1). Sepsis will afflict 1.7 million adults in America, and result in 270,000 each year, attributing a huge economic burden to the US health care system (2). The activation of pattern recognizing receptors (PRRs) of immune cells by pathogen-associated molecular patterns (PAMPs) and damage-associated molecular patterns (DAMPs), results in an immune response to intruders (3, 4). Initiation of inflammation following infection is important to eradicate infection, while excessive inflammation can cause tissue injury, multi-organ dysfunction, and death in sepsis. A clear understanding of this pathophysiology will assist in removing the barriers of developing effective medications to treat sepsis.

Sialic acid-binding immunoglobulin-type lectins (Siglecs) are a group of receptors expressed on multiple types of immune cells. Siglecs are classified into two groups. The first group of Siglecs conserved in human and mouse are Siglec-1, -2, and -4 known as sialoadhesin, CD22, and myelin-associated glycoprotein (MAG) respectively. Siglec-15 is also a member of this group. The second group of Siglecs are known as CD33-related Siglecs. This group is rapidly evolving and comprises 11 members in humans, and 5 members in the mouse (5, 6). The CD33 family of human Siglecs includes CD33 or Siglec-3, -5, -6, -7, -8, -9, -10, -11, -12, -14, and -16. The mouse CD33 orthologs are designated by letters, which include CD33 (Siglec-3), Siglec-E, -F, -G, and -H. The number of extracellular immunoglobulin (Ig) domains in Siglecs varies among different Siglecs, for example Siglec-1 has 17 Ig domains, while Siglec-3 (CD33) and -H, each having only 2 Ig domains (5). Murine Siglec-G’s human ortholog is Siglec-10. Here, we focus on the immunoregulatory functions of Siglec-G during sepsis, because of the ubiquitous presence of sialic acid, the ligand of Siglec-G, on cell membranes, membrane receptors, and glycoproteins. Siglec-G is most highly expressed in B-1a cells, but is also present on conventional B-2 cells, where Siglec-2 is predominantly expressed (6, 7). Dendritic cells (DC), macrophages, and T lymphocytes also express Siglec-G (6, 7). It contains five extracellular Ig-like domains. The N-terminal Ig-like domain, which is similar to the variable domain of Ig (V-set domain), contains the sialic acid binding motif. The other parts of Siglec-G include a transmembrane region as well as an intracellular tail with three different tyrosine-based motifs: i) one immunoreceptor tyrosine-based inhibitory motif (ITIM), ii) one ITIM-like domain, and iii) a Grb-2-binding site (6, 8). Siglec-G binds to sialic acid moieties on B cell receptor (BCR), thereby controlling BCR-mediated signal transduction. Deficiency of Siglec-G causes altered BCR signaling which leads to increased Ca^++^ influx, increased nuclear factor of activated T-cells, cytoplasmic 1 (NFATc1) expression, and decreased Src homology region 2 domain-containing phosphatase 1 (SHP1) levels in B-1a cells (7, 8). Siglec-G knockout mice have been found to have expanded populations of B-1a cells in the peritoneal cavity as well as elevated circulatory natural IgM. Overall, a skewed repertoire of secreted natural IgM implicating an altered IgM function has been detected in Siglec-G knockout mice (9, 10). Siglec-G also downregulates NF-κB activation in B-1a cells, which influences B-1a cell proliferation and survival (10, 11). Thus, Siglec-G is a crucial component of B-1a cell signaling and development. In myeloid cells, Siglec-G acts as a negative regulator of DAMPs. CD24 directly binds multiple different DAMPs, which then in turn binds to Siglec-G forming a trimeric assembly, serving to repress the inflammatory responses to DAMPs (12). Thus, preserving Siglec-G’s function protects mice from inflammation. The expression of Siglec-G at its mRNA level was increased in LPS-induced sepsis, although the protein level of Siglec-G and its expression in various immune cells during sepsis are unknown (13). By contrast, a recent report has shown that irradiation can cause reduced expression of Siglec-G at its protein level in antigen presenting cells (APCs), which leads to DAMP-mediated inflammatory responses (14). Since irradiation often causes sepsis by disrupting the intestinal barrier resulting in a leak of intestinal flora into the circulation, the findings of deceased expression of Siglec-G in APCs in the irradiation model could be mimicked with a polymicrobial sepsis model. Given the importance of Siglec-G in regulating the immune response, the expression of Siglec-G in various immune cells under inflammatory conditions should be studied.

B-1a cells are a unique subpopulation of B cells with profound immunoregulatory properties. B-1a cells have innate-like functions, playing a critical role in the initial defense against invading pathogens by secreting natural Abs (IgM) that help to protect the host from acute infection as well as lower bacterial load (15). B-1a cells either spontaneously or following infection produce the anti-inflammatory cytokine IL-10 in bulk amounts (16). B-1a cells also release granulocyte-monocyte colony-stimulating factor (GM-CSF) (17), which governs emergency myelopoiesis as well as causing B-1a cells to release more IgM in an both an autocrine and paracrine manner to protect the host against infection (17, 18). Conversely, B-1a cells also produce IL-3, IL-17, and TNF-α, which exhibit pro-inflammatory roles in sepsis (15, 19). B-1a cell-mediated protective outcomes have been reported in bacterial sepsis, pneumonia, and viral infection (15). In this review, we discuss the role of Siglec-G in B-1a cells and other cells to regulate BCR- and PRR-mediated pathways to control inflammation in sepsis. We also discuss the therapeutic interventions by targeting Siglec-G in sepsis. Unveiling the potential role of Siglec-G in B-1a cells and other cells is important for understanding the pathobiology of sepsis and its therapy.

## Rationale

Siglec-G is expressed in a wide range of immune cells such as B-1a cells, myeloid cells, and T cells (6, 8, 14). B-1a cells have been shown to provide a survival benefit as well as ameliorate end organ damage in sepsis (15, 16). Although the role of Siglec-G in B-1a cells in the context of sepsis has not been studied before, given the evidence of Siglec-G-mediated anti-inflammatory role in CD24 expressing myeloid cells (20), it is speculated that B-1a cells which also express CD24 and Siglec-G could play similar anti-inflammatory role in sepsis (21). Myeloid cells play a pivotal role in innate-immunity in sepsis. Following infection, these cells respond to PAMPs and become activated to release pro-inflammatory cytokines and DAMPs including high-mobility group box 1 (HMGB1), extracellular cold-inducible RNA-binding protein (eCIRP), and histones (4). The transcription factor NF-κB induces the expression of these pro-inflammatory molecules (22). Given the importance of Siglec-G in controlling DAMP-mediated inflammation by inhibiting NF-κB, the immunoregulatory role of Siglec-G in sepsis is prominent and needs to be further discussed to dissect its mechanism of action in sepsis. Sialic acids are nine-carbon sugars attached to glycopeptides and glycolipids found in the circulation and on cell surfaces. *N*-glycolylneuraminic acid (Neu5Gc) and *N*-acetylneuraminic acid (Neu5Ac) are the two sialic acids most commonly found in mammalian cells. Sialic acids are attached to glycans *via* α2-3, α2-6 or α2-8 linkages (5, 6). The specific orientation of these linkages is often crucial for recognition by the sialic acid binding proteins expressed on mammalian cells. Siglec-G binds sialic acid moieties in a cis (same cell) or trans (adjacent cells) acting manner (9), which widens the scope of Siglec-G’s role in sepsis as increased cell to cell interaction is evident in sepsis. Given the increased expression of several glycoproteins which are enriched in sialic acids in inflammatory diseases (23), there seems to be a possibility that sialic acid contents could be increased in sepsis. This in turn may serve to activate Siglec-G to turn on the immunoregulatory mechanism in sepsis. The expression of Siglec-G was shown to be significantly upregulated in immune cells upon stimulation with lipopolysaccharide (LPS) (13), implicating Siglec-G’s impact in sepsis. Since the deficiency of Siglec-G could play a beneficial role in sepsis, here the increase of Siglec-G in their model could exhibit detrimental outcomes in sepsis (13). Since the sepsis pathophysiology and etiologies are complex and diverse, relying on a particular study finding may not reflect real clinical scenarios. Collectively, these strong scientific premises led us to focus on Siglec-G’s role in B-1a cells and beyond in sepsis.

## Sialic Acid-Binding Immunoglobulin-Type Lectin-G Contributes to Host Protection in Sepsis

**Figure 1:**
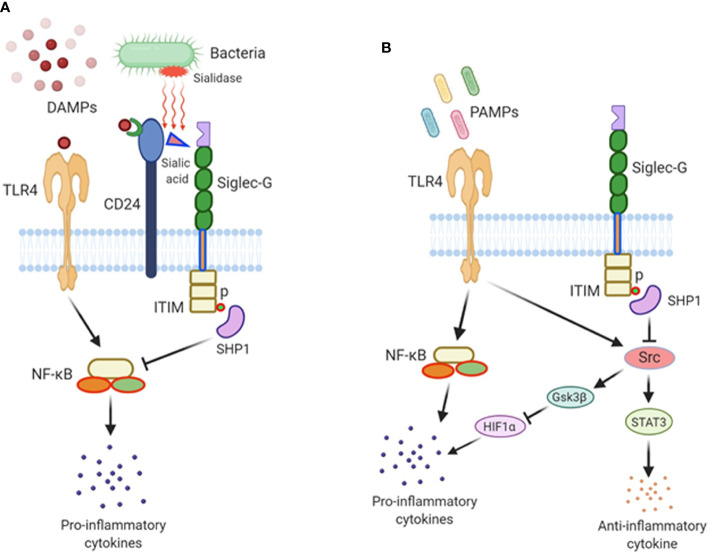
The role of Siglec-G in sepsis. **(A)** Damage-associated molecular patterns (DAMPs) are released from damaged cells during sepsis and recognized by TLR4, fueling inflammation. DAMPs also bind to CD24, and this di-molecular complex further binds to Siglec-G through CD24’s sialic acid moiety to form a tri-molecular complex. Siglec-G leads to SHP1 activation which downregulates NF-κB activity and pro-inflammatory cytokine production. In polymicrobial sepsis, bacterial sialidases remove sialic acid residue from CD24, abrogating the CD24-Siglec-G interaction, thus enhancing the inflammatory process. **(B)** Siglec-G regulates PAMPs-TLR4-mediated signaling during sepsis. Siglec-G inhibits Src activation through the activation of SHP1 *via* Siglec-G’s ITIM domain. Src inhibits TLR4-induced inflammatory cytokines and increases the expression of IL-10 by activating STAT3. In Siglec-G deficient macrophages due to less recruitment of SHP1, Src is activated at its optimal level possibly through TLR4 pathway. Src then promotes HIF1α degradation through activating GSK3β. HIF1α is critical in inducing LPS-induced inflammatory response. As such, Src-induced degradation of HIF1α results in less pro-inflammatory cytokines production upon LPS stimulation in Siglec-G deficient immune cells. Thus, Siglec-G coordinates TLR4-induced pro- and anti-inflammatory cascades in sepsis.

Siglec-G is expressed in B-1a cells, as well as in myeloid and lymphoid cells to play immunoregulatory functions (6, 12). Since these cells play a crucial role in sepsis, Siglec-G’s role in sepsis is critical. There exists a large body of evidence demonstrating the key role of NF-κB activation in sepsis. Studies have demonstrated that NF-κB inhibitors protect animals from sepsis (24, 25). NF-κB is constitutively activated in Siglec-G^-/-^ B-1a cells (11). In DCs, Siglec-G hinders DAMPs effects on NF-κB activation (12). In myeloid cells, Siglec-G causes SHP2 and Cbl-dependent ubiquitylation and proteasomal degradation of RIG-I resulting in a dampening of the type I IFN response (26). Given the decreased activation of NF-κB and type-I IFN by Siglec-G, sepsis-induced hyperinflammation can be controlled. The direct role of Siglec-G in polymicrobial sepsis was first identified by using Siglec-G^-/-^ mice, which showed increased susceptibility to sepsis-induced death (20). Similarly, the Siglec-G’s interacting molecule CD24^-/-^ mice showed increased mortality in sepsis. Corresponding to the increased mortality in the mutant mice, the levels of IL-6, MCP-1, and TNF-α were sharply elevated. Compared to wild-type counterparts, the lung, kidney, and liver of CD24^-/-^ and Siglec-G^-/-^ mice showed severe hemorrhage, venous congestion, and necrosis (20). The CD24-Siglec-G interaction has been shown to be a crucial negative regulator of inflammation in sepsis. Sialidases are a potent virulence factor produced by many different invading pathogens, and sialic acid-based pattern recognition is a cardinal feature of Siglec-G. Therefore, bacterial sialidases may exacerbate sepsis by CD24 desialylation. Treatment of CD24 protein with recombinant sialidases from three different bacteria, *S. pneumoniae*, *C. perfringens*, and *V. cholerae* dramatically reduced Siglec-G’s binding with CD24 and therefore exacerbated HMGB1 and HSP70 induced inflammation in sepsis (20). Following sepsis, there is a marked increase in sialidase activity, which disrupts CD24-binding to Siglec-G leading to uncontrolled inflammation (**Figure 1A**). CD24 is not the only molecule that contains sialic acids and also the Siglec-G is not the only receptor that binds to sialic acids to become affected by the bacterial sialidases, there could be a number of molecules which contain sialic acids, binding to other Siglecs, and also become desialylated by bacterial sialidase. As such, the strategy and the findings as made by Chen et al. (20) focuses only on the CD24 and Siglec-G, given the fact that the deficiency of either CD24 or Siglec-G causes detrimental outcomes in sepsis. These findings further shed light on the avenues of identifying other sialic acid containing ligands and Siglecs that become affected by bacterial sialidase to exacerbate sepsis.

Siglec-G^-/-^ B-1a cells possess a constitutively active BCR-mediated intracellular signal transduction pathway, leading to an increase in B-1a cell number as well as elevated levels of natural IgM (7). In context of B-1a cell produced natural IgM’s role, the protective outcomes of Siglec-G^-/-^ mice have been demonstrated in atherosclerosis (27). Natural IgM capable of binding OxLDL are significantly increased in the plasma and peritoneal cavities of Siglec-G^-/-^ mice (27). Consistent with the neutralizing functions of OxLDL-specific IgM, Siglec-G^-/-^ mice were shown to have protection from OxLDL-induced sterile inflammation. Despite the increased blood IgM levels, the Siglec-G^-/-^ mice are more susceptible to peritoneal bacterial infections as noticed in CLP-induced sepsis (20). Blood and peritoneal lavage fluid cultures yielded more bacterial colonies in Siglec-G^-/-^ mice than wild-type controls. Although bacterial load in WT and Siglec-G^-/-^ was unaffected, the sialidase activity in the serum of Siglec-G^-/-^ mice after sepsis was increased compared to WT mice in sepsis, which might lead to detrimental outcomes in septic Siglec-G^-/-^ mice (20).

The immunoregulatory function of B-1a cells is altered by the presence of various bacterial proteins. Porin of *Shigella dysenteriae* type 1, an enteric bacteria, induces the proliferation of B-1a cells, but it dramatically reduces Siglec-G on B-1a cells (28). The porin-induced reduction of Siglec-G expression by B-1a cells is characterized by marked elevation of CD69 and CD40 expression, indicating activation of these cells subsequent to the down regulation of Siglec-G. As a result of cell activation, porin skews IL-10 producing/competent B-1a cells toward pro-inflammatory cytokines producing/responsive cells (28). Thus, bacterial infection shifts B-1a cell phenotype towards pro-inflammatory through a decreased expression of Siglec-G.

## Human Sialic Acid-Binding Immunoglobulin-Type Lectin-10 Mimics the Function of Murine Sialic Acid-Binding Immunoglobulin-Type Lectin-G

To describe how the findings of murine Siglec-G can be matched with its human orthologue Siglec-10 during inflammation, at first the interaction of human CD24 and human Siglec-10 was determined. Sialidase treated recombinant human CD24 when combined with recombinant human Siglec-10 did not interact with each other, confirming the importance of sialic acid on CD24 for binding to Siglec-10 to transduce the downstream immunoregulatory signal, controlling DAMP-mediated inflammation (20). This result with human Siglec-10 suggests that the findings on murine Siglec-G to ameliorate inflammation can be transferable to human sepsis. In addition to this study, murine Siglec-G’s function was shown to be connected with its human orthologue Siglec-10 in a microbial sepsis model (29). In their study, Stephenson et al. showed that C. jejuni flagella can promote an anti-inflammatory axis *via* glycan-Siglec-10 engagement. They revealed that pseudaminic acid residues on the flagella contributed to IL-10 expression in dendritic cells. They also identified the ability of both viable C. jejuni and purified flagellum to bind to Siglec-10. *In vitro* infection of Siglec-10 overexpressing cells with pseudaminic acid residues containing flagella resulted in increased IL-10 expression in a p38-dependent manner. Detection of Siglec-10 on intestinal dendritic cells added further credence to the notion that this novel interaction may contribute to immune outcome during human infection.

## Contradictory Role of Sialic Acid-Binding Immunoglobulin-Type Lectin-G in Pamp-Mediated Sepsis

A recent study explained Siglec-G’s role in endotoxemia in a different angle, where they suggested that Siglec-G deficiency attenuated the LPS-TLR4-induction of pro-inflammatory cytokines, and augmented the expression of anti-inflammatory cytokine IL-10 at both acute and immunosuppressive phases of sepsis (13). This study explains the impact of Siglec-G on PAMPs-induced inflammation, as our previous discussion mainly explained Siglec-G’s role in DAMPs-mediated inflammation. This non-classical function of Siglec-G was mediated though its regulation of activation of proto-oncogene tyrosine-protein kinase (Src). Src activation is inhibited by Siglec-G through the recruitment and activation of the tyrosine phosphatase SHP1. Src inhibits TLR4-induced inflammatory cytokines and promotes anti-inflammatory cytokine IL-10 production. In Siglec-G^-/-^ macrophages due to less recruitment of SHP1, Src is activated at its optimal level. Src then promotes hypoxia inducible factor 1α (HIF1α) degradation through activating GSK3β. HIF1α is critical in inducing LPS-induced inflammatory response. As such, in Siglec-G^-/-^ macrophages, Src-induced degradation of HIF1α results in less pro-inflammatory cytokines production upon LPS stimulation. Conversely, it has been shown that Src interacts with and phosphorylates STAT3, which leads to increased expression of IL-10, given the fact that STAT3 positively regulates IL-10 expression. Thus, Siglec-G orchestrates TLR-induced inflammation, which leads to the conclusion that the treatment of acute and chronic inflammatory diseases may be achievable through the blockade of Siglec-G or the activation of Src by inhibiting pro-inflammatory cytokines and inducing anti-inflammatory cytokine production (**Figure 1B**). Treatment of septic patients with anti-inflammatory therapy may result in a protracted immunosuppressive phase. However, the blockade of Siglec-G (activation of Src) while the patient is in the hyper-inflammatory phase or the activation of Siglec-G (blockade of Src) while in the hypo-inflammatory phase of sepsis may prove a useful treatment adjunct.

By comparing the findings of two contradictory studies with Siglec-G-deficient mice in sepsis, we found Li et al. showed that Siglec-G deficiency ameliorates hyperinflammation and immune collapse in endotoxemia (13), on the other hand, Chen’s study revealed that Siglec-G^-/-^ mice showed detrimental outcomes in CLP-induced sepsis (20). Towards delineating the mechanism, Chen et al. determined the role of Siglec-G in attenuating DAMP-mediated inflammatory responses in sepsis, while Li et al. demonstrated that after LPS challenge, Siglec-G^−/−^ mice produced less IL-6 and TNFα, more IL-10, and had an improved survival rate compared to Siglec-G^+/−^ mice. However, in contrast to Li et al’s findings, another study demonstrated Siglec-G’s immunomodulatory role in DAMP-mediated, but not PAMP-mediated inflammation (12). They showed that the survival rates between WT and Siglec-G^-/-^ mice in endotoxemia model did not differ between WT and Siglec-G^-/-^ mice (12). Considering these two opposite findings with Siglec-G in DAMP- and PAMP-mediated inflammation, targeting Siglec-G for its activation or modulation may not always be beneficial in sepsis. Thus, additional studies may be required to prove Siglec-G’s definitive role in DAMP- and PAMP-mediated inflammation.

## Sialic Acid-Binding Immunoglobulin-Type Lectin-G-Mediated Signal Transduction in B-1A Cells

Mature murine B lymphocytes are broadly categorized into three subsets. Follicular (FO) B cells, marginal zone (MZ) B cells, and B-1 cells. Follicular B cells, which are also called B-2 cells, are the most prevalent subset. B2 cells are found in the lymphoid follicles of the spleen and lymph nodes. FO B cells, upon interaction with CD4^+^ T helper cells, can differentiate into short lived plasma cells, or can form a germinal center (GC) and become long lived plasma cells or memory B cells (30). MZ B cells possess attributes of both naïve and memory B cells. They are located in the marginal sinus of the spleen where they are exposed to pathogens and particulate antigens (31).

B-1 cells were first described in 1983 by Hayakawa et al. (32). These cells are found in the peritoneal and pleural cavities, as well as the spleen. B-1 cells are characterized as B220^lo^, CD19^+^, CD23^-^, CD43^+^ (15, 33). B-1 cells were first termed Ly-1^+^ (murine) or Leu-1^+^ (human) due to their expression of the aforementioned surface markers (32). These surface markers have since been renamed CD5. Further research has determined that the presence of CD5 has come to distinguish B-1a (CD5^+^) cells from B-1b cells (CD5^-^). B-1 cells originate from distinct hematopoietic progenitor cells. B-1a cells are unique in their ability to release repertoire skewed polyreactive natural antibodies. These natural antibodies serve as a first line of defense by eliminating many different types of pathogens (15, 34). Whereas the B-1b cells exhibit adaptive antibody responses to pneumococcal polysaccharide type 3 (PPS-3) and are essential for long-term protection against *S. pneumoniae* infection (35). B-1a cells are the most prolific producers of IL-10 by B lymphocytes (16, 36). The first phase of B-1 cell development occurs in fetal development and continues into neonatal life. These early B-1 cells largely contributes to the adult B-1 cell compartment, as B-1 cells are capable of self-renew throughout the lifetime of the organism (34).

**Figure 2:**
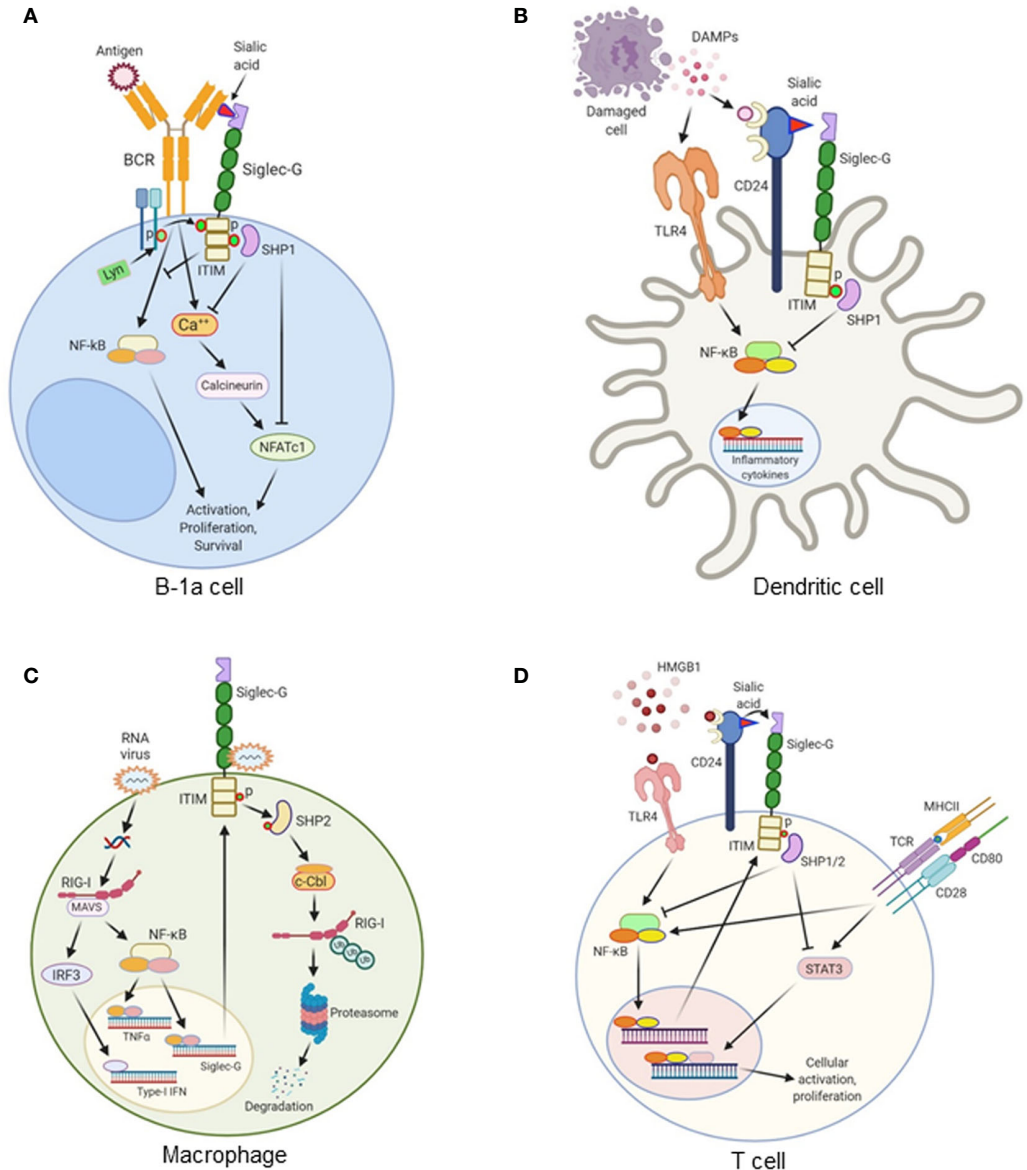
The role of Siglec-G in various immune cells. **(A)** Antigen recognition by the B cell receptor (BCR) on B-1a cells triggers a Ca^++^ influx and activates NFATc1 *via* calcineurin. This leads to the survival, proliferation, and activation of B-1a cells. Siglec-G is recruited to the antigen-activated mIgM, where it binds sialic acid moieties on the mIgM. The ITIM domain of Siglec-G is phosphorylated by Lyn kinase, resulting in the recruitment of the tyrosine phosphatase SHP1, inhibiting both mIgM-induced Ca^++^ signaling and NFATc1 expression. Siglec-G also inhibits NF-κB activation and regulates NF-κB-dependent B-1a cells activation, proliferation, and survival. **(B)** Damage-associated molecular patterns (DAMPs) like HMGB1, HSP70, and HSP90 are released from damaged cells during sterile or infectious inflammation. DAMPs are recognized by TLRs, which subsequently activates NF-κB and increases the expression of proinflammatory cytokines. CD24 expressed on the surface of dendritic cells (DCs) binds DAMPs. CD24 also binds to Siglec-G, thereby forming a tri-molecular complex. This leads to the activation of SHP1 through Siglec-G-mediated signaling. SHP1 inhibits DAMPs-mediated activation of NF-κB, ultimately leading to the decreased production of pro-inflammatory cytokines. **(C)** In macrophages, double-stranded RNA viruses enter into the cells and recognize RIG-1 coupled with MAVS receptors and activate the transcription factors NF-κB and IRF3. NF-κB increases the expression of Siglec-G as a positive feedback loop mechanism, and increases the expression of pro-inflammatory cytokines. IRF3 increases the expression of type I IFNs. RNA viruses also bind to Siglec-G and activates its associated SHP2, which causes Cbl-dependent ubiquitylation and proteasomal degradation of RIG-I, leading to the suppression of IFNβ expression through a negative feedback loop. **(D)** HMGB1 induces TCR-, and CD28-mediated T cell activation, proliferation, and cytokine production through TLR4-NF-κB- and STAT3-dependent pathway. HMGB1 increases Siglec-G expression through TLR4-NF-κB pathway. HMGB1 binds to CD24, and CD24 binds to Siglec-G. This tri-molecular complex activates SHP1 and SHP2 to inhibit NF-κB and STAT3 activation, leading to the decreased cellular activation and proliferation.

Siglec-G serves as a negative regulator of BCR (7, 8, 10). Siglec-G is associated, to some extent, with membrane-bound (m)IgM through binding to its sialic acid motif. The binding of antigen to mIgM results in the recruitment of more Siglec-G to the activated receptor. This interaction takes place *via* α2-3 or α2-6-linked sialic acids present on mIgM. The recruitment of Siglec-G to the antigen-activated mIgM, results in tyrosine phosphorylation of ITIMs on Siglec-G, most likely by Lyn. The phosphorylation of ITIMs results in the recruitment of the tyrosine phosphatase SHP1, which inhibits both mIgM-induced Ca^++^ signaling and NFATc1 expression (7, 10). Despite having some disputes, Siglec-G has also been shown to regulate NF-κB activation in B-1a cells through BCR, as Siglec-G deficiency causes activation of this transcription factor (11). Siglec-G-deficient mice have been found to express significantly higher amounts of cytosolic phosphorylated IκB as well as nuclear accumulation of P65 in peritoneal lavage samples. Since NF-κB governs the expression of a number of pro- and anti-inflammatory cytokines in sepsis (24), Siglec-G’s regulation on NF-κB activation may have an impact on these cytokines production by B-1a cells in sepsis. Thus, in B-1a cells Siglec-G works mainly on BCR signaling to regulate NFATc1 and NF-κB pathway (**Figure 2A**).

## Sialic Acid-Binding Immunoglobulin-Type Lectin-G Deficiency on B-1A Cells

Signal transduction of the BCR is crucial for initiating B-cell response. Siglec-G knockout mice were found to have extensively enlarged B-1a cell populations (7, 8). Interestingly, the B-1b and B-2 cells were not affected by the loss of Siglec-G in these mice, suggesting the inhibitory role of Siglec-G is much more crucial in B-1a cells. The absence of Siglec-G resulted in higher calcium mobilization by B-1a cells upon BCR stimulation. This increase in calcium signaling has been attributed to the lack of SHP1 recruitment resulting in increased BCR signaling (7). Siglec-G deficient B-1a cells were noted to undergo lower rates of apoptosis and found to have an extended life span (10, 37, 38). This decrease in apoptosis could be a result of greater expression of NFATc1 in Siglec-G-deficient B-1a cells. In addition to NFATc1 activation, studies have also showed that activation of Siglec-G results in the down regulation of NF-κB, which limits the size of B-1a cell lineage (11). Activation of NF-κB is required for the self-renewal of the B-1a cells in the peritoneal cavity and spleen of the Siglec-G^-/-^ mice. Despite the fact that NF-κB plays an important role in B-1a cells and as opposed to this data by Ding et al. (11), Jellusova et al. did not find and difference in the activation of NF-κB in Siglec-G deficient mice (37). The discrepancies between these two studies could be due to the fact that Ding et al. mostly performed experiments with lysates from total unseparated cell populations from peritoneal washouts; whereas Jellusova et al. chose to study purified B-1a cells (11, 37). In addition, the possibility that the genetic background plays a role in these two studies may not be excluded because one group used BALB/c background, while the other group used C57BL/6. In addition to these facts, the Siglec-G’s impact on NF-kB in B-1a cells may be not as promising as myeloid cells, as a recent study demonstrate the atypical response of B-1 cells to BCR ligation (39). In this report it is mentioned that peritoneal B-1a cells manifest unusual signaling characteristics that distinguish them from B-2 cells (39). These include the failure of BCR engagement to trigger NF-κB activation and DNA replication given the central role for phosphatase activity. B-1a cells are unable to induce NF-κB or proliferate after BCR cross-linking due to increased phosphatase abundance or activity. This phosphatase abundance and/or activity may be the result of unique B-1a cell characteristics such as increased levels of HSP70 and/or constitutive secretion of IL-10. Furthermore, constitutively active Lyn also plays a negative regulatory role in B-1a cells.

B-1a cells have been shown to secrete natural antibodies in mice. These antibodies are primarily IgM isotype (40, 41). As Siglec-G^-/-^ mice had more B-1a cells, their IgM titers in serum were also higher than WT mice (7, 27). Interestingly, there were no differences in the production of other isotypes of Ig when compared to WT controls. Moreover, the Siglec-G^−/−^ mice had a greater number of IgM-secreting cells both in the bone marrow and in the spleen. The number of IgG-secreting cells was not found to be different. No differences were found in the numbers and structure of B cell follicles in Siglec-G^−/−^ spleen and in WT spleen when stained for sialoadhesin and anti-IgM. Normal B cell numbers were noted in the marginal zone as well. However, Siglec-G^−/−^ sections were noted to show greater extrafollicular IgM-secreting plasma cells compared to WT sections (7, 42, 43).

Intriguingly, the Siglec-G^−/−^ B-1a cells were noted to have an altered BCR repertoire compared with wild-type B-1a cells (37). The BCR repertoire and the VDJ composition of Igs of Siglec-G^−/−^ B-1a cells is similar to the Abs produced by adult bone marrow-derived B cells rather than canonical fetal liver-derived B-1a cells. This suggests that differentiation of precursors into the B-1a cell population is altered in Siglec-G-deficient mice. Holodick et al. showed that B-1a cells from older mice have higher amounts of N-region additions compared to B-1a cells from younger mice (44), which could cause altered naïve B-1a cells- and natural IgM-mediated protective outcomes during infection. Further studies are needed to define the phenotypic and functional aspects of B-1a cells derived from wild-type vs Siglec-G-deficient mice.

Upon BCR stimulation, B-1 cells generally do not have as robust of a response as B-2 cells (45, 46). Anti-IgM antibodies cause weakened Ca^++^ signaling and cellular proliferation in B-1a cells as compared to B-2 cells (8, 46). On the other hand, B-1a cells have a pre-activated phenotype demonstrating increased expression of cell surface activation markers and show pre-activation of signaling pathways such as the ERK and NFATc1 (46, 47). Therefore, besides BCR-mediated signaling as it is weakly responsive in B-1a cells, studying other pathways in B-1a cells needs to be further emphasized. A growing body of literature has elucidated the function of Siglec-G on the regulation of BCR signaling, while the studies on Siglec-G’s effects on innate immune sensors like PRRs were mostly overlooked. Since B-1a cells produce both anti- as well as pro-inflammatory mediators (15), studies on the impact of Siglec-G on the expression of pro- and anti-inflammatory mediators in B-1a cells in sepsis would be of great interest.

## Sialic Acid-Binding Immunoglobulin-Type Lectin-G-Mediated Signal Transduction in Myeloid Cells

Siglec-G is expressed in myeloid cells, i.e., DCs and macrophages (6, 48), where it plays an essential role. The first report of the effects of Siglec-G in myeloid cells was determined in DCs, in which it was shown to attenuate DAMPs-, but not PAMPs-mediated inflammation in sterile liver injury (12). This addresses the pivotal question of how our immune system distinguishes between DAMPs and PAMPs signals (49). The glycoprotein receptor CD24 is expressed at high levels in hematopoietic cells and plays a critical role in Siglec-G mediated immunoregulatory function (12). It is unlikely that CD24 and Siglec-G act directly on hepatocytes as there is currently no evidence showing these cells express Siglec-G. DCs respond to HMGB1, a putative DAMP, and express both CD24 and Siglec-G. In sterile liver injury, HMGB1 levels are elevated in the liver to further aggravate inflammation and liver injury. However, HMGB1-mediated liver injury was greatly reduced and the mice were protected from lethal injury by the presence of Siglec-G in hematopoietic cells (12). CD24 has been found to only bind with Siglec-G or -10, but not with other Siglecs. CD24 also binds to the B-box motif of HMGB1. As such, CD24, Siglec-G and HMGB1 form a tri-molecular complex. This generates a downstream signaling through the ITIM motif of Siglec-G to activate SHP1, a known negative regulator of NF-κB, thereby inhibiting HMGB1-mediated pro-inflammatory cytokine production (12). Both LPS and HMGB1 induce nuclear translocation of NF-κB p65 in wild-type DCs; however, in CD24 or Siglec-G-deficient DCs, HMGB1 can induce higher levels of p65 nuclear translocation compared to LPS. They also provided survival data to mimic clinical outcomes which showed worse outcomes in CD24^-/-^ mice in the liver ischemia model, while the survival rates were not affected between WT and CD24^-/-^ or Siglec-G^-/-^ mice after LPS injection. This reflects the fact that CD24 and Siglec-G negatively regulate immune responses to HMGB1, HSP70, and HSP90, but not to LPS and poly I:C (12). These findings suggest that the CD24-Siglec-G pathway will preferentially dampen the response to DAMPs without having an impact on PAMP signaling by selective repression of NF-κB activation. In addition to a nuclear DAMP like HMGB1, DCs also respond to cytoplasmic DAMPs such as HSP70 and HSP90 through a TLR-dependent pathway (12, 49). CD24 has been found to bind both HSP70 and HSP90. Similar to HMGB1, the association of Siglec-G with HSP70 and HSP90 requires CD24. CD24^-/-^ and Siglec-G^-/-^ DCs have been shown to produce significantly higher amounts of IL-6 and TNF-α in response to recombinant HSP70 and HSP90 compared to wild-type DCs. These data show that CD24 and Siglec-G are crucial surface proteins involved in the negative regulation of DCs response to several different DAMPs (**Figure 2B**).

Besides DCs, the effects of Siglec-G-CD24 pathway in macrophages for inhibiting DAMPs-mediated inflammation is also evident. Siglec-G expression is upregulated on macrophages in an NF-κB- or retinoic acid-inducible gene-I (RIG-1)-dependent manner following infection by RNA viruses such as vesicular stomatitis virus (VSV), but not DNA viruses or bacteria (26). The upregulation of Siglec-G results in SHP2 and Cbl-dependent ubiquitylation and subsequent proteasomal degradation of RIG-I as well as the suppression of the interferon β (IFNβ) response (**Figure 2C**). Due to the detrimental effects of type I IFN in murine polymicrobial sepsis, Siglec-G-mediated inhibition of the production of type I IFN by macrophages in sepsis could be beneficial. Interestingly, the suppression of VSV-triggered IFNβ *via* Siglec-G does not require CD24 nor is it affected by sialidase treatment of the macrophages, suggesting it is sialic acid-independent. Degradation of sialic acid moieties may not be required as the Siglec-G receptors appear to retain phosphorylation of their ITIM domains and subsequently recruit SHP2 after VSV infection (26).

Prior reports reveled that Siglec-G attenuates DAMPs, but not PAMPs induced inflammatory responses. However, a recent study determined the direct interactions between various Siglecs and PRRs (50). Human Siglec-5/9 and mouse Siglec-3/E/F have been shown to bind to some TLRs. Mouse Siglec-G was not shown to interact directly with any TLRs tested. Correspondingly, the deletion of Siglec-E resulted in an augmented dendritic cell response to all microbial TLR ligands tested, while Siglec-G deletion did not show evidence of an altered response. TLR4 activation results in the translocation of neuraminidase 1 (Neu1) to the cell surface, disrupting the interaction between TLR4 and Siglec-E. Conversely, treatment with the sialidase inhibitor Neu5Gc2en resulted in preservation of TLR4-Siglec E/F interactions. Preservation of the sialic acid-TLR-Siglec interaction in mice lacking Neu1 or treated with the sialidase inhibitor Neu5Gc2en resulted in protection from endotoxemia (50). These findings suggest that sialidase mediated de-repression or Siglec mediated repression of TLR function could result in positive feedback of TLR activation. During bacterial infection, both PAMPs and DAMPs fuel inflammation. At first, PAMPs induce tissue damage, causing a release of DAMPs to further exaggerate inflammation. Even though Siglec-G does not exhibit direct interaction with PAMPs, it recognizes DAMPs through a tri-molecular complex with CD24 and inhibits inflammation (12).

## The Role of Sialic Acid-Binding Immunoglobulin-Type Lectin-G in T Cells

T cells express Siglec-G, and its expression is increased upon stimulation with HMGB1 (51). While Siglec-G plays a pivotal role in B cell development and proliferation, it has no influence on T cell development or differentiation at homeostasis. Under normal conditions, Siglec-G^-/-^ mice demonstrate no difference in numbers or distribution of naive, central memory, effector memory, or Treg cells. Nonetheless, Siglec-G^–/–^ T cells demonstrated significantly augmented rates of cellular proliferation in the presence of HMGB1 and anti-CD3/CD28 Abs when compared with Siglec-G^–/–^ T cells lacking stimulation from DAMPs (51). Siglec-G regulates the HMGB1**’**s effects on T cells through ITIM and SHP1 and SHP2 pathways (**Figure 2D**). STAT3 is activated in HMGB1 treated Siglec-G^-/-^ T cells, while the lymphocyte-specific protein tyrosine kinase is not. Interestingly, in the presence of HMGB1, Siglec-G^–/–^ T cells had elevated markers of activation, but had no difference in the expression of exhaustion markers such as programmed cell death protein-1 (PD-1), T cell immunoreceptor with Ig and ITIM domains (TIGIT), or lymphocyte-activation gene 3 (Lag3) when compared with wild-type T cells (51). Therefore, Siglec-G/ITIM signaling is required to control DAMP-mediated increase of the activation signaling or the exhaustion markers. These findings shed light on the pathobiology of graft-versus-host disease (GVHD) in which a T cell-autonomous role is critical for modulating the severity of the T cell-mediated immunopathology (51). GVHD was ameliorated by treatment with the CD24Fc fusion protein, enhancing the Siglec-G signaling in donor T. As such, Siglec-G may serve as a potential therapeutic target in the treatment of GVHD (14, 51, 52).

Siglec-G inhibits antigen-presenting cells (APC) and cytotoxic T lymphocytes (CTL) interaction, thereby inhibiting CTL proliferation by inhibiting the formation of MHC class I peptides (53). In APC such as DCs, phagosome-expressed Siglec-G recruits the phosphatase SHP1, which dephosphorylates the NADPH oxidase component p47(phox) and inhibits the activation of NOX2 on phagosomes. The inhibition of NOX2 results in reduced cross-presentation to CTLs by hydrolyzing foreign antigens and diminishing antigen presentation *via* MHC class-I peptides (53). Therefore, Siglec-G inhibits DC cross-presentation by impairing such complex formation, and this finding adds insight into the regulation of cross-presentation in adaptive immunity.

## The Role of Sialic Acid-Binding Immunoglobulin-Type Lectin-G on Leukocyte Migration During Inflammation

During sepsis, leukocytes are recruited to inflamed tissues through their interaction with adhesion molecules present on endothelial cells (3, 54). Vascular adhesion protein-1 (VAP-1) is a glycoprotein expressed on inflamed endothelium with two crucial roles role: it has enzymatic activity resulting in the oxidation of primary amines and also serves as an adhesion molecule that is involved in leukocyte trafficking to sites of inflammation. Siglec-10 (the human homologue of murine Siglec-G) serves as a leukocyte ligand for VAP-1 (55). The interaction between Siglec-10 and VAP-1 results in a greater production of hydrogen peroxide, indicating that Siglec-10 serves as a substrate for VAP-1. Moreover, Siglec-10-VAP-1 interaction seems to mediate lymphocyte adhesion to the endothelium. This interaction may serve to modify the inflammatory microenvironment *via* production of various enzymatic end products (55). Since myeloid cells express Siglec-10 in human or -G in mice, elucidation of its role in enhancing the migration and infiltration of mononuclear cells in the inflamed tissues during sepsis is of interest.

Sepsis often causes acute kidney injury (AKI), which is characterized by excess accumulation of leukocytes (neutrophils and monocytes) into the kidneys to cause inflammation and tissue damage (56). As opposed to the above reports on VAP-1-Siglec-10-mediated lymphocyte migration during inflammation, a recent study demonstrates an indirect effect of Siglec-G as a negative regulator of leukocyte migration into the kidney tissues during AKI (57). After AKI, there is an increase in circulating and kidney B cells, particularly a B220^low^ subset. These B220^low^ B cells, presumably B-1 cells, produce the chemokine CCL7, which promotes infiltration of neutrophils and monocytes into the injured kidney parenchyma. Siglec-G^-/-^ mice, which have increased numbers of B220^low^ innate B cells, had increased levels of CCL7, augmented recruitment of neutrophils and monocytes to the kidney, and more severe AKI. A reduction in myeloid cell infiltration into the was noted after CCL7 blockade in AKI (57). These finding suggest that B cells may play a crucial role in the early sterile inflammation in AKI by producing leukocyte-recruiting chemokines. Siglec-G, by regulating the production of CCL7 by B-1 cells, inhibits leukocyte accumulation in kidneys in sepsis. As a result of the effects of Siglec-G in augmenting or inhibiting leukocyte migration, additional studies should be undertaken to gain a more detailed understanding of the mechanism of leukocyte migration during sepsis.

## Modulating Sialic Acid-Binding Immunoglobulin-Type Lectin-G for Therapeutic Approaches in Sepsis

It has been shown that bacterial sialidases can remove sialic acid residues from CD24, resulting in an abrogation of the CD24-Siglec-G interaction in mice or CD24-Siglec-10 interaction in humans, leading to an augmentation of the inflammatory process (20). Therefore, inhibition of sialidase will result in a protection in mice from bacterial sepsis in a CD24-Siglec-G-dependent fashion. Studies on bacterial mutants lacking sialidase have confirmed the importance of this enzyme as a virulence factor in sepsis and demonstrate the importance of Siglec-G in controlling the inflammatory responses (20). Siglec-G participates in the attenuation of inflammatory responses for both pathogen and non-pathogen mediated signals. Two sialidase inhibitors, 2,3-dehydro-2-deoxy-N-acetylneuraminic acid (Neu5Ac2en) and 2,3-dehydro-2-deoxy-N-glycolylneuraminic acid (NeuGc2en) have been synthesized and their efficacy in treating polymicrobial sepsis has been tested (20). A combination of the two inhibitors resulted in complete inhibition of the sialidase activity in the sera of septic mice. This ultimately reflected decreased levels of multiple inflammatory cytokines and significantly reduced mortality in sepsis. The dependence on the CD24 and Siglec-G genes demonstrates a specificity of the inhibitors and suggests that the protection is likely achieved by preserving Siglec-G-CD24 interaction. Since septic patients are often treated with antibiotics, sialidase inhibitor therapy was administrated in conjunction with antibiotics. While antibiotic treatment alone had some impact on survival, the addition of sialidase inhibitors resulted in significantly increased survival in septic mice. These results indicate that treatment with sialidase inhibitors in conjunction with antibiotics have cellular implications exceeding potential inhibition of bacterial growth (20).

In light of the above promising finding of the treatment with the sialidase inhibitors in sepsis, a recent study developed a nanoparticle coated with di(α2→8) *N*-acetylneuraminic acid (NANA), which mimics sialic acid, the natural ligand for Siglec-E, which is predominantly expressed in hematopoietic cells such as macrophages and neutrophils (58). Treatment with the nanoparticle resulted in an augmentation of anti-inflammatory activity in culture as well as improving survival in multiple different mouse models, two generalized septic and one pulmonary injury. This nanoparticle has been shown to be effective in human macrophages and in an ex vivo model of human lung injury. In this study, we noticed that these NANA-coated NP provided protection in both LPS- and CLP-driven sepsis models, which was mediated through the Siglec-E pathway (58). However, two prior reports showed opposing outcomes with Siglec-G, in which Siglec-G^-/-^ mice provided protection against CLP (20), but did not in LPS-induced model (13). These contradictory results in Siglec-G^-/-^ mice may raise the possibility that since these NPs blocked both the CLP- and LPS-driven models of sepsis, promoting Siglec-G’s function with sialic acid mimics may not be involved in the action of sialic acid mimics as Siglec-G deficiency has opposing impacts upon these two models. Spence et al. identified that IL-10 induced Siglec-E expression and α2,8 NANA-NP further augmented the expression of IL-10 (58). Indeed, the effectiveness of the nanoparticle depended on IL-10. However, in the report by Li et al. showed that Siglec-G deficient mice or the macrophages had less TNFα and IL-6, but more IL-10 levels in the serum or supernatants compared to WT mice after treatment of mice or cells with LPS (13). Considering IL-10 as the key player in NANA-NP treated and Siglec-G deficient conditions to control LPS induced inflammation, further studies should be performed in mice strains and their age and gender matched conditions, because Li et al’s findings on the beneficial outcomes of Siglec-G^-/-^ mice in LPS induced survival did not correlate with the findings of Chen et al. (12), which showed no statistically significant beneficial outcomes in the survival rates between WT and Siglec-G^-/-^ mice following LPS treatment. Moreover, prior studies have shown that HMGB1, CD24 and Siglec-G form a trimolecular complex that induces Siglec-G-mediated Shp1 activation to downregulate TLR4-mediated inflammation (20). Therefore, the treatment with NANA-containing NPs may bind Siglec-G and block the interaction of CD24 with Siglec-G, which may exhibit altered function other than that of Siglec-G-CD24-HMGB-1-mediated regulatory function.

Given the fact that Siglec-G binds to α2,3-linked or α2,6-linked sialic acid (α2,3Sia or α2,6Sia), the α2,8 NANA-NP may not bind to Siglec-G as efficiently as Siglec-E. But, this novel strategy can be further implemented to create α2,3Sia or α2,6Sia NP to specifically target Siglec-G in sepsis. These findings encourage further research to identify sialic acid containing endogenous peptides or glycoproteins to confer protection in microbial sepsis and cell damage-induced sterile inflammation. Potential therapeutic approaches and outcomes by modulating Siglec-G in sepsis are shown in **Table 1**.

**Table 1:** Therapeutic outcomes by modulating Siglec-G in sepsis.

Therapeutic approaches	Inflammatory/injury outcomes	Survival outcomes	References
Siglec-G^-/-^ mice in CLP model	Siglec-G^-/-^ mice significantly increased the levels of IL-6, MCP-1, and TNFα in the serum; serum bacterial loads were same in WT vs Siglec-G^-/-^ mice; lungs, liver, and kidney injuries were aggravated in Siglec-G^-/-^ mice.	Survival rates of Siglec-G^-/-^ mice in both with or without antibiotic treated conditions were declined.	(12, 20)
Siglec-G^-/-^ mice in endotoxemia (LPS) model	Siglec-G^-/-^ mice showed decreased levels of IL-6 and TNFα and increased levels of IL-10 in the serum; Siglec-G^-/-^ mice had less severe lung injury.	Siglec-G^-/-^ mice showed improved survival outcomes in endotoxemia model.	(13)
Sialidase inhibitors: Neu5Ac2en (AC) and Neu5Gc2en (GC)	AC + GC treatment decreased serum levels of IL-6, MCP-1, and TNFα, but had no effect on reducing blood bacterial contents.	AC + GC treatment improved survival rates in both with or without antibiotic treated conditions.	(20)
Sialidase inhibitor:Neu5Gc2en	Inhibited IL-6 and TNFα levels in the serum.	Improved survival rates in LPS and *E. Coli* induced sepsis.	(50)
Nanoparticles decorated with Siglec ligand, di(α2*→*8) *N*-acetylneuraminicacid (α2,8 NANA-NP)	Decreased TNFα and increased IL-10 levels in the serum; decreased neutrophils in the lungs; reduced lung inflammation and injury.	Improved survival rates in both LPS- and CLP-induced sepsis models.	(58)

## Conclusion and Future Directions

Siglec-G-mediated immunoregulatory functions in sepsis have been extensively studied in the context of myeloid cells. Given the significance of B-1a cells in sepsis, elucidation of Siglec-G’s role in B-1a cells altering its phenotype and function in sepsis is vital. Recently, the phenotype, ontogeny, and function of human B-1a cells have been discovered (21). Therefore, determining the expression and function of Siglec-10, the human orthologue of murine Siglec-G in human B-1a cells in sepsis patients are important. The outbreak of COVID-19 in the US and worldwide has resulted in fatal outcomes for these patients. A recent perspective proposed treating COVID-19 with B-1a cells to mitigate the cytokine storm and eliminate viral loads by B-1a cell-produced IL-10 and natural IgM, respectively (59). Considering Siglec-G’s immunomodulatory functions in B-1a cells and myeloid cells, studies on Siglec-G could be a promising area to elucidate the pathophysiology and therapeutic interventions of COVID-19.

Genetic polymorphism studies in humans provide unique opportunities to understand human biology and the mechanisms of diseases. These genetic studies have shown correlations between various human diseases and Siglec genes, for example the CD33 polymorphism is associated with Alzheimer’s disease (5). Therefore, studying the genetic polymorphism of human Siglec-10 may benefit reinforcement by independent genetic replication or mechanistic studies in disease pathogenesis. Sialic acid is crucial in maximizing the phagocytic activity of cells (60). Treatment of polymorphonuclear cells with bacterial neuraminidases completely abolishes stimulation of phagocytic activity. Given the importance of membrane sialic acid for stimulation of phagocytosis, Siglec-G’s role as a cis- and trans-acting receptor to enhance phagocytosis should be elucidated in sepsis, because impaired efferocytosis is a hallmark of sepsis (3). In chronic inflammatory and autoimmune diseases, B-1a cells are found to be detrimental (61). Studies have shown that peritoneal B-1a cells proliferate and collect in inflamed joint tissue with upregulated receptor activator of nuclear factor kappa-β ligand (RANKL) expression during collagen-induced arthritis development in mice (62). Since Siglec-G regulates BCR signaling and helps maintain a normal B-1a cell pool, its role in B-1a cell mediated chronic inflammatory diseases could be promising. The impact of Siglec-G in immune cells with a wide range of DAMPs stimulation should be assessed. Extracellular CIRP was recently described as a novel DAMP (63). Identification of the role of eCIRP on Siglec-G in B-1a cells and beyond, in terms of the production of pro-inflammatory mediators in sepsis, will help broaden our understanding of the pathophysiology of sepsis. This review demonstrates the immunoregulatory functions of Siglec-G in B-1a cells, myeloid, and lymphoid cells in sepsis, which ultimately improves our understanding of the pathophysiology of sepsis for developing novel therapeutics against this deadly disease.

## Author Contributions

WR and MA wrote the manuscript. WR and PW reviewed and edited the draft. MA and PW conceived the idea and supervised the project. All authors contributed to the article and approved the submitted version.

## Funding

This study was supported by the National Institutes of Health (NIH) grants R35GM118337 (PW) and R01GM129633 (MA).

## Conflict of Interest

The authors declare that the research was conducted in the absence of any commercial or financial relationships that could be construed as a potential conflict of interest.

## Abbreviations

BCR, B cell receptor; DAMPs, damage-associated molecular patterns; eCIRP, extracellular cold-inducible RNA-binding protein; GM-CSF, granulocyte-macrophage colony-stimulating factor; GVHD, graft-versus-host disease; HMGB1, high-mobility group box 1; IFNβ, interferon β; IRF3, interferon regulatory factor 3; ITIM, immunoreceptor tyrosine-based inhibition motif; NFATc1, nuclear factor of activated T-cells, cytoplasmic 1; OxLDL, oxidized low-density lipoprotein; PAMPs, pathogen-associated molecular patterns; PD-1, programmed cell death protein-1; PRRs, pattern recognizing receptors; RANKL, receptor activator of nuclear factor kappa-β ligand; RIG-I, retinoic acid-inducible gene-I; SHP1, Src homology region 2 domain-containing phosphatase 1; Siglec-G, Sialic acid-binding immunoglobulin-type lectin-G; TIGIT, T cell immunoreceptor with Ig and ITIM domains; VAP-1, vascular adhesion protein-1.
